# Theory of mind in patients with mild cognitive impairment: A systematic review

**DOI:** 10.3389/fpsyg.2022.994070

**Published:** 2022-10-18

**Authors:** Lucia Morellini, Alessia Izzo, Martino Ceroni, Stefania Rossi, Giorgia Zerboni, Laura Rege-Colet, Elena Biglia, Leonardo Sacco

**Affiliations:** ^1^Faculty of Biomedical Sciences, Università della Svizzera italiana, Lugano, Switzerland; ^2^Neuropsychological and Speech Therapy Unit, Neurocenter of Southern Switzerland, Ente Ospedaliero Cantonale (EOC), Lugano, Switzerland

**Keywords:** theory of mind, mild cognitive impairment, social cognition, systematic review, tasks

## Abstract

The focus of this systematic review was to collect and align studies which analyze the functionality of theory of mind (TOM) in patients with mild cognitive impairments (MCI). Specifically, we identified 20 papers published between 2012 and 2022 which met inclusion criteria. Papers search, selection, and extraction followed the PRISMA guidelines. In order to summarize data from the papers, we used a narrative synthesis approach. Results in 18 of these 20 papers show that theory of mind (TOM) is impaired in all types of MCI patients—regardless of different etiology and diagnostic criteria. Only 2 out of 20 reported no significant differences in TOM performance between MCI patients and healthy control subjects. The review additionally aimed to bundle the variety of the type of tasks used by the author to assess multiple domains of TOM. This heterogeneity does not allow us to make a comprehensive comparison between the results, so we suggest the need to align the results using the same type of tests and TOM assessment. In the end, our work highlights the 2 neuropsychological studies which confirm more of our results; due to the objective approach adopted to investigate this topic, we suggest exploring this point of view more in future research.

## Introduction

The ability to represent and attribute mental states to self and others—such as beliefs, emotions, desires, and knowledge—is called Theory of Mind (TOM) (Premack and Woodruff, 1978).

The first investigation about TOM dates to Premack and Woodruff (1978) and affirms that humans have the capacity to assume what others feels, believe, want, etc. and for those reasons, they infer states undeclared or not observable in a direct way and use these inferences to predict the behavior of others and their own, in an anticipatory way.

Theory of mind permits us to understand that others can have different values, beliefs, and feelings from ours. This kind of comprehension is a infinitely helpful in social interactions, because it allows us to make inferences on others' mental states and to deduce their behaviors (Premack and Woodruff, 1978).

TOM is not an innate ability; for this reason, Wimmer and Perner (1983) led studies in order to understand how children learn the representation of others' mental states and how they become able to differentiate them from the real world and their own. Thanks to the ideation of “false belief task” we know now that children acquire TOM abilities at around 4 years old. More recent studies, affirm that using more accurate methodology (for example, monitoring of gaze direction) children < 4 years old can also succeed in TOM tasks (Clements and Perner, 1994; Baillargeon et al., 2010).

In order to develop TOM abilities, there are several skills that each child should acquire first. The main skills are concept of attention, understanding others' intentions, and imitating others (Westby and Robinson, 2014; Bosco et al., 2017).

First, Baron-Cohen (1991) considers attention as a key concept and the real starting point to developing theory of mind, in particular regard for joint attention (when two people direct the attention to the same point/object, often pointing at it for more accuracy of direction).

Second, regarding intentionality, we mean the understanding that others' behaviors are directed toward a specific goal, which is determined by personal belief and desires (Dennett, 1983). Understanding that other's actions are driven by personal impulse, means being aware that everyone has their own desires, and so knowing how, attribute mental states. This ability is already developed in 2 year olds, not only in humans, but also in chimpanzees and orangutans (Call and Tomasello, 1998; Luchkina et al., 2018).

Last, the concept of imitating others means understanding that they have personal desires and beliefs. The first two concepts are fundamental to be able to copy other people, because it means realizing that people direct attention (to something or someone) in a motivated and personal way (drivers by personal desires).

As mentioned previously, already around 4 years old, children begin to consider the feelings and thoughts of others. Some researchers identified 5 phases of the development of TOM associated to a specific task to overcome (Wellman and Liu, 2004; Peterson et al., 2012).

Every phase consists of the ability to understand (Wellman and Liu, 2004; Peterson et al., 2012):

1. Wanting: Means realizing that others act in various ways in order to achieve different goals, influenced by their desires.
2. Thinking: Means realizing that others' actions are based on what they think could happen in a certain situation. The same situation could have different points of view because they are influenced by subjective beliefs.
3. Knowing: Seeing leads to knowing consists of the ability to recognize that others have different access to information. Often extra information is necessary to explain to other people what they have not seen or experienced.
4. False Beliefs: This concept refers to the ability to comprehend that other people may have incorrect beliefs that are not close to reality.
5. Hidden Feelings: Involves the ability to understand that sometimes others' emotions are masked. It means that it could happen that displayed emotions are different from real feelings.

TOM, according to several studies (Rossetto et al., 2018), is a multidimensional construct that includes different level of complexity; the attribution of intentions passed through the first order level of attribution, while the attribution of emotions concerns the second order level of attribution.

It is important to underline that TOM is often distinguished between cognitive and affective (Wang and Su, 2013): the ability to understand beliefs, intentions, and thoughts refers to cognitive TOM, differentiated from affective TOM that consists of thinking about their own or others' emotions and affect.

In this regard, there are several tests to assess TOM, such as the Reading the Mind in the Eyes Test (specific for affective TOM; Baron-Cohen et al., 2001), the Story Based Empathy Task (both for cognitive and affective TOM; Dodich et al., 2015) and the Faux Pas Recognition Test (specific for cognitive TOM; Stone et al., 1998) (for more complete information, see Table 2).

Different research showed that TOM can be impaired in several neurodegenerative disorders, such as in Alzheimer's Disease (as reported by Morese et al., 2018; Kessels et al., 2021; Morese and Palermo, 2022), Parkinson's Disease (Rossetto et al., 2018; Adenzato et al., 2019; Morese and Palermo, 2020), Frontotemporal Dementia—behavioral variant (Adenzato et al., 2010; Poletti et al., 2012; Orso et al., 2022), but also in cognitive disorders without dementia, such as Mild Cognitive Impairment (MCI). In this regard, several studies reported lower performance in many TOM tasks in MCI patients, compared to healthy populations (Baglio et al., 2012; Poletti and Bonuccelli, 2013; Moreau et al., 2015; Adenzato et al., 2019; Orso et al., 2022).

Mild Cognitive Impairment (MCI) is, by definition, considered a prodrome of dementia, a transitional phase between healthy aging and dementia (Anderson, 2019; Breton et al., 2019). In this phase patients have an increased risk of developing dementia in the following years, but their daily functioning is not yet impaired (Petersen, 1996a; Smith et al., 1996). The term MCI was first defined by Petersen et al. (1997) in order to describe this phase of transition, in which patients, as said before, do not meet criteria for dementia (Albert et al., 2011).

Also, in the DSM V (American Psychiatric Association, 2013), we can find a diagnostic category called “mild neurocognitive disorder (mild-NCD), to describe MCI. DSM V Diagnostic criteria meet the previously reported criteria (Petersen et al., 1997; Albert et al., 2011) and underline a mild cognitive decline in one or more domains (for example, executive function) that do not interfere with independence in daily activities, and it cannot be explained by a delirium context or by mental disorders (for example, depression or schizophrenia) (American Psychiatric Association, 2013).

Petersen (2016b) also provides us a distinction between 2 types of MCI: amnestic MCI (aMCI) and non-amnestic MCI (naMCI). Respectively, aMCI is related to deficits only in the memory domain, while naMCI is associated to deficits in single or multiple cognitive domains (for example, executive functions, language, memory, visuospatial abilities etc.), typically naMCI is prodromal of Alzheimer's disease (Albert et al., 2011).

Another specification that is important to report in MCI diagnosis is pathologic etiology on which cognitive decline depends, the main are: Alzheimer's Disease (AD-MCI), Parkinson's Disease (PD-MCI), Frontotemporal dementia (FTD-MCI), vascular disease (VaMCI), and Lewy body disease (MCI-LB) (Albert et al., 2011; Litvan et al., 2011; American Psychiatric Association, 2013).

Based on what was previously described, we performed a systematic review of the current literature to align and understand the current state of science on this topic; the aim of the present review is to deepen theory of mind in MCI patients. Since there is still a lot of ambiguity, this review can provide the opportunity to explore weaknesses and limitations on this argument and it can be a starting point for future research.

### The goal of present review

This work is led by the necessity to align and collect studies that analyze social cognition in patients with MCI, in particular, regarding Theory of Mind. Generally, we know that social cognition is impaired in neuropsychological diseases (such as, frontotemporal dementia, Alzheimer's) (Morese et al., 2018; Palermo et al., 2020; Dodich et al., 2021), so we could expect that results in TOM tasks in patients with MCI might be insufficient compared to the control group. At the same time, we could also expect that TOM performance in MCI patients might be less impaired than performance in patients with severe neuropsychological disease (such as Alzheimer's).

## Methods

The present systematic review was conducted according to the Preferred Reporting Items for Systematic Reviews and Meta-Analyses (PRISMA, Moher et al., 2009). The method is currently available in the Open Science Framework (OSF, https://osf.io/hn7mc/).

### Eligibility criteria

The focus of this systematic review was to collect studies which analyze the functionality of theory of mind (TOM) in patients with mild cognitive impairments (MCI).

The inclusion criteria were as follows:

- Studies must include populations with a diagnosis of mild cognitive impairment (MCI), evaluated by standardized diagnostic criteria of Petersen et al. (1997, 1999); Petersen and Negash (2008); Petersen (2016b) or DSM V criteria (American Psychiatric Association, 2013) or Winblad et al. (2004) or Albert (PD-MCI—Albert et al., 2011) or every cognitive impairment—without dementia—diagnosed with a validated cognitive test; for example, Dementia Rating Scale (DSR) (Mattis, 1988) or PD-MCI (Litvan et al., 2011).
- The medium age of the sample must be 60 years old, either male or female.
- We included all types of MCI: amnesic MCI (aMCI), non-amnesic MCI (naMCI), Parkinson's MCI (PD-MCI), Alzheimer's MCI (AD-MCI), Vascular MCI (VaMCI).
- We included studies that evaluated the domains of social cognition “theory of mind (TOM)”.
- Studies must include at least one clinical cognitive measurement for the social cognition domain analyzed (TOM).

The exclusion criteria were:

- Articles not in English were excluded.
- Meta-analysis, systematic reviews, single case studies, or other studies with a small sample (e.g., studies with < 10 participants) or only qualitative measurements, comments, books conference papers, letters, theses, and all studies not peer-reviewed were excluded.

## Information sources

### Search strategy

The search of the present study was conducted across Pubmed and Medline databases. For the MCI search strategy, we used the following terms: “MCI” OR “mild cognitive impairment”. The keywords were combined with the domain of social cognition: “theory of mind (TOM”) to produce the results.

### Study selection

We only considered studies limited to humans and with a limited range period from January 2012 to May 2022. The reason behind this choice was that before 2012 we did not find significant studies which met our inclusion criteria (for example, before 2012 were published only studies which analyzed cognitive components and not social cognitive components related to MCI patients).

**Figure 1:**
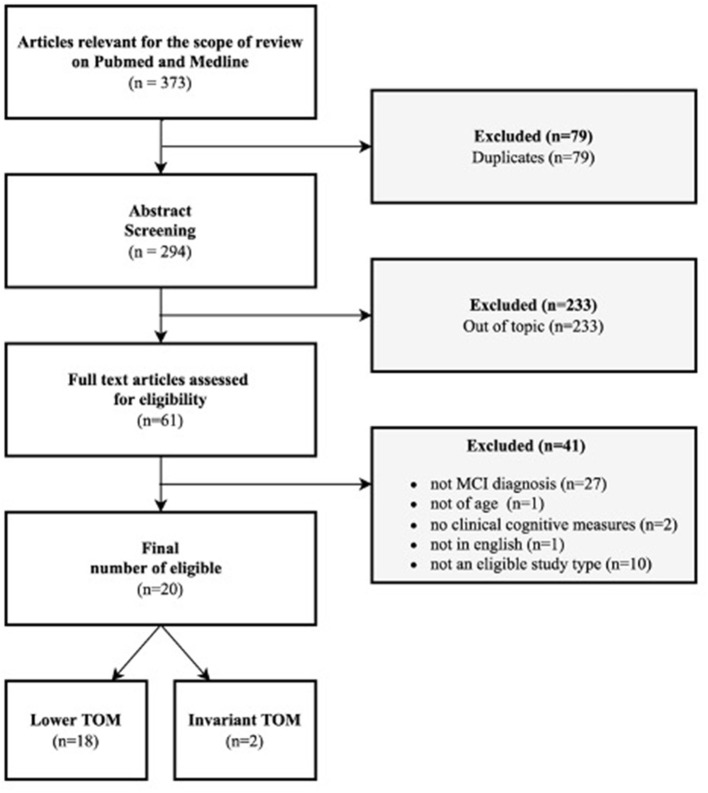
Flow diagram.

Moreover, meta-analysis, other systematic reviews, case studies, qualitative studies, or every study with a very small sample and without quantitative measurements were excluded from the present review. Initially, the papers included in the selection were 373, and then we excluded 79 duplicates. Reading title and abstract, from 294 articles we excluded 233 other articles out of the topic. Only 61 papers were considered eligible for the scope of the present review. Those 61 papers were further analyzed by reading the complete text, to discover if they respected inclusion criteria. At this point, another 41 articles were excluded because 27 did not have an MCI diagnosis (in line with inclusion criteria), 1 did not have a sample >60 years of age, another 2 did not have a clinical cognitive measurement to assess the domain selected for the present review (TOM), 1 was not in English and the last 10 were review or meta-analysis (as descripted previously in inclusion criteria). In the end, 20 articles were included in our review (see Figure 1).

## Results

Overall, 20 studies were included in our review. Those studies examine the domain of theory of mind (TOM) (*N* = 20). As shown in the Table 1, in line with literature, most studies (*N* = 18) reported lower performance in Theory of Mind (TOM) than the control groups. Only 2 studies did not report significant differences.

**Table 1:** Theory of mind (*N* = 20).

**References**	**Participants**	**Age range (MCI)**	**Summary of relevant findings**	**Cognitive measures adopted**
Baglio et al. (2012)	16 aMCI and 15 healthy controls	58–77	aMCI subjects reported an overall worse performance than HC to all ToM tasks, except for the Deceptive Box Task. HC subjects had increased activity in the right superior temporal gyrus and the TP cortex during the Reading the Mind in the Eyes test.	The Eye-Direction Detection; The Deceptive Box Task; The look-prediction and say-prediction task; A selection from the strange stories. The Reading the Mind in the Eyes (RMET)
Maki et al. (2013)	A total of 31 young normal controls, 104 aged normal controls, 42 patients with amnesic mild cognitive impairment, and 30 patients with mild AD	69–79	Summary, both sarcasm and metaphor were significantly lower in all groups respect YNC. Respectively, sarcasm was worse in all groups except from YNC. Metaphor was worse in aMCI and M-AD than both control groups (but the aged one, was worse than the younger)	Metaphoric and Sarcastic Scenario Test
Poletti and Bonuccelli (2013)	20 aMCI and 20 healthy controls	65–78	The lower RME performance of aMCI patients provide evidence that aMCI may be associated with difficulties in tasks of ToM	Reading the Mind in the Eyes Test (RMET)
Gaudreau et al. (2013)	31 aMCI and 33 healthy controls	68–81	The authors demonstrated a lower performance on a verbal irony comprehension task for aMCI individuals compared with those in the HC group	A French adaptation of the task created by Winner et al. (1998) was used in this study to evaluate verbal irony comprehension
Gaudreau et al. (2015)	30 MCI, 30 healthy controls	60–83	participants with MCI have second-order mentalizing difficulties compared to HC subjects	Mentalizing abilities (Combined Stories Test) and verbal irony comprehension (SSICT)
Moreau et al. (2015)	20 MCI and 25 healthy controls	64–84	MCI patients presented difficulties inferring another person's beliefs about reality and attributing knowledge to them in a situation of real-life interaction	The false belief task (Nosy-neighbor) The referential communication tasks
Rossetto et al. (2018)	16 aMCI, 14 Parkinson disease, 18 Healthy Control	67–80	aMCI group had lower performance than healthy control group on Reading the Mind in the Eyes Test and Strange Stories Task. aMCI group scored lower on the total score of the Yoni Task, compared with healthy control	Deceptive Box Task Look Prediction and Say Prediction Task Reading the Mind in the Eyes Test (RMET) Strange Stories Task Italian Version of the Yoni Task (computerized)
Adenzato et al. (2019)	20 PD-MCI and 20 Healthy Control	57–74	The patients with PD-MCI had significantly worse accuracy than the HC did in the Reading the Mind in the Eyes Test. The patients with Parkinson Disease MCI had significantly worse accuracy and Reaction Times in the Intention Task than the HC	Attribution of Intentions task Reading the Mind in the Eyes Test (RMET)
Formica et al. (2020)	24 AD, 24 FTD, 10 MCI (Same numbers of caregivers for each patient)	60–73	Overall, if caregivers have low results in TOM tasks, patients also have poorer TOM results (there's a correlation between TOM'S performance and caregivers' distress)	Reading Mind in the Eyes Test (RMET) Stories Empathy Task (SET)
Rossetto et al. (2020)	30 patients with aMCI, 21 healthy controls	72–81	aMCI group underperformed the HC group only in the advanced ToM tasks. There were time changes in some subcognitive, cognitive and affective ToM dimensions in aMCI subjects.	Deceptive Box task Reading the Mind in the Eyes Test (RMET) A selection of four stories Strange Stories task (SS task)
Yildirim et al. (2020)	18 adults with early stage AD, 31 adults with MCI, 32 adults with SCI.	56–73	The MCI group displayed deteriorated performance on RMET but not on FPR. ToM performance was significantly related to episodic memory and verbal fluency within the overall sample.	Reading the Mind in the Eyes Test (RMET) Faux-Pas Recognition test (FPR)
Eramudugolla et al. (2021)	132 patients with MCI, 23 patients with dementia, 1272 healthy controls	73–77	Participants with MCI and dementia showed poorer RMET performance than cognitively normal participants. Participants with MCI and dementia reported reduced social network size.	Reading the Mind in the Eyes Test (RMET)
Kessels et al. (2021)	31 adults with aMCI, 29 patients with AD, 45 healthy controls	69–82	aMCI and AD patients performed worse on answering questions requiring the ability to infer the thoughts and feelings of others in theory of mind ability.	Story comprehension Task
Michaelian et al. (2021)	19 adults with aMCI, 24 adults with naMCI,	53–73 51–74	aMCI subgroup revealed a significant association between poorer ToM performance and reduced functional connectivity between the bilateral temporal pole and the left lateral temporal cortex; between the right TempP and the dorsal medial prefrontal cortex; and between the left and right TempP.	Reading the Mind in the Eyes Test (RMET)
Tsentidou et al. (2021)	41 adults with VRF, 44 adults with MCI, 24 healthy controls	63–77	MCI group showed lower performance on ToM tasks, than HC.	The Awareness of Social Inference Test (TASIT)
Valera-Bermejo et al. (2021)	37 patients with MCI, 9 patients with mild stage probable AD dementia, 34 healthy controls	68–82	Social cognition scores were associated with lower connectivity of the default-mode network with the prefrontal cortex. Default-mode network includes highly associated with TOM performance (Mars et al., 2012; Li et al., 2014)	Reading the Mind in the Eyes Test (RMET) Stories Empathy Task (SET)
Giguère-Rancourt et al. (2022)	12 patients with PD-MCI	65–75	Patients have difficulties to understandand and guess the intentions (cognitive ToM) of their interlocutor, the greater the distress experienced by caregivers. PD-MCI's cognitive ToM difficulties seem to be linked to caregiver feelings of distress	Faux Pas Recognition test (FPR)
Orso et al. (2022)	25 patients with MCI-AD, 24 patients with bvFTD, 40 healthy controls	75–86	The hubs of the ToM network were identified in frontal regions in both bvFTD and MCI–AD patients. MCI–AD patients performed worse than HC. MCI patients showed a poor performance only in the RMET.	Reading the Mind in the Eyes Test (RMET) Reading the Mind in the Eyes Test Region of Interests (RMET-ROIs)
Dodich et al. (2015)	12 Alzheimer's disease, 20 bv fronto temporal dementia, and 15 aMCI; 65 healthy controls	67–79	Dementia (bvFTD and AD) showed impaired performances in all subconditions of SET. aMCI and healthy controls did not differ in the results	Story based empathy task
Funkiewiez et al. (2012)	22 Fronto Temporal Degeneration patients, 22 patients with Alzheimer's disease or amnesic mild cognitive impairment (aMCI), and 30 healthy control subjects	53–88	aMCI patients performances were not significantly different from HC in Theory of mind task (Faux Pas recognition test)	Faux Pas Recognition test (FPR)

Specifically, a study conducted by Baglio et al. (2012) on a sample of 16 amnesic MCI (aMCI) vs. healthy controls (HC), using a comprehensive battery of cognitive tasks (The Eye-Direction Detection; The Deceptive Box Task; The look-prediction and say-prediction task; A selection from the strange stories; The Reading the Mind in the Eyes test during fMRI—see the article for further and complete information), showed that aMCI subjects reported an overall worse performance than HC to all TOM tasks, except for the Deceptive Box Task (Perner et al., 1987; Happé, 1994; Sullivan et al., 1994; Baron-Cohen et al., 1995; Antonietti et al., 1999; Mazzola and Camaioni, 2002; Snowden et al., 2003; Sullivan and Ruffman, 2004; Castelli et al., 2011).

Another study (Maki et al., 2013), evaluated TOM with a different task (Metaphoric and Sarcastic Scenario Test—Adachi et al., 2004) on a sample of 31 young normal control (YNC), 104 aged normal controls (ANC), 42 patients with aMCI, and 30 patients with mild AD (M-AD). The results showed that both sarcasm and metaphor were significantly lower in all groups with respect to YNC. Respectively, sarcasm was worse in all groups except YNC. The metaphor was worse in aMCI and M-AD than in both control groups (but the aged one was worse than the younger). They used this task because sarcasm and metaphor could be compromised aspects in people with cognitive impairment. The most common task used to assess TOM is “The Reading the Mind in the Eyes test” (RMET; Baron-Cohen et al., 2001), for example, Poletti and Bonuccelli (2013) used this task on a sample of 20 aMCI vs. 20 HC, described a lower RMET performance of aMCI sample. Likewise, Gaudreau et al. (2013) with a similar sample (31 aMCI vs. 33 HC), used a test to evaluate verbal irony comprehension (Winner et al., 1998) and reported lower performances in the aMCI group. Also, Gaudreau et al. (2015) adopted a similar test for irony and mentalizing abilities (Achim et al., 2012) and reported the same results as the previous (difficulties in the second order of TOM in MCI sample).

Additional ways to evaluate TOM are “the False Belief Task” and the “Referential Communication Task” (Samson et al., 2007; Champagne-Lavau et al., 2009) used by Moreau et al. (2015) on a sample of 20 MCI; this study showed MCI patient had difficulties in others' beliefs and knowledge in a situation of real-life interaction compared to HC.

Rossetto et al. (2018), utilizing a large battery of tests (see Table 1), has discovered that a sample of 16 aMCI performed worse than the HC group on most tasks adopted. Adenzato et al. (2017) evaluated a sample of PD-MCI (*n* = 20) and showed a worse performance both in RMET and Attribution of Intention Task (Baron-Cohen et al., 2001; Adenzato et al., 2017).

Yildirim et al. (2020) conducted a study using the RME Test and the FPR Test (Faux Pas Recognition Test) (Baron-Cohen et al., 2001; Gregory et al., 2002) and discovered that the MCI group (*n* = 31) displayed deteriorated performance on RMET but not on FPR.

Rossetto et al. (2020) with a similar battery of tests used in a previous study (Rossetto et al., 2018), discovered that the aMCI group (*n* = 30) unperformed the HC only in the advanced TOM task (RMET and Strage Stories Task—Baron-Cohen et al., 2001; Happé, 1994; Mazzola and Camaioni, 2002). The RME Test (Baron-Cohen et al., 2001) was also used by Eramudugolla et al. (2021) on a sample of MCI (*n* = 132) and dementia patients (*n* = 23); the study displayed that MCI and dementia's groups showed a poorer RMET performance than HC group.

In line with the study of Moreau et al. (2015), Kessels et al. (2021), using the Story Comprehension Task (Oosterman et al., 2011) reported that aMCI (*n* = 31) and AD (*n* = 29) groups had difficulties inferring the thoughts and feelings of others.

Along the same line, Tsentidou et al. (2021) tested a sample of 31 adults with aMCI, 29 patients with AD, and 45 healthy controls and they discovered, using TASIT (The Awareness of Social Inference Test—McDonald et al., 2003), that performance on TOM tasks were worse in aMCI than in HC.

Two authors, included in the review, have reported a possible factor that could influence low results in TOM tasks (Formica et al., 2020; Giguère-Rancourt et al., 2022). The first of those studies included a sample of 10 MCI patients and the second study included a sample of 12 PD-MCI (with the same number of respective caregivers), and showed a possible correlation between low TOM performance in MCI patients and caregiver's distress. They assessed patient and caregivers with affective and cognitive TOM tasks (RMET, SET and FPR; Stone et al., 1998; Baron-Cohen et al., 2001; Gregory et al., 2002; Dodich et al., 2015), and both reported that MCI TOM difficulties seems to be linked to caregivers' distress.

Three studies adopted a neuropsychological approach, investigating the neural substrate of theory of mind.

First, Michaelian et al. (2021), on a sample of 43 adults with MCI (aMCI, *n* = 19; naMCI, *n* = 24) used RMET (Baron-Cohen et al., 2001) to assess TOM and functional magnetic resonance imaging to investigate the alterations in resting-state functional connectivity within the default mode network (DMN). The authors discovered that the aMCI group revealed a significant association between poorer TOM performance and reduced functional connectivity between the bilateral temporal pole (TempP) and the left lateral temporal cortex. The same phenomenon occurs between the right TempP and the dorsal medial prefrontal cortex and a decreased functional connectivity was reported also between the left and right TempP.

Valera-Bermejo et al. (2021) investigated TOM performance on a trial of 37 patients with MCI (mild stage with probable dementia, *n* = 9; healthy controls, *n* = 34) using the RME Test and the SET (Baron-Cohen et al., 2001; Dodich et al., 2015) showed that TOM scores were correlated with lower connectivity of the DMN with the prefrontal cortex.

In the end, also the most recent study of the present review (Orso et al., 2022), agrees with the previous studies. In fact, it is reported that MCI-AD patients (*n* = 25) showed a poorer performance in the RMET compared to HC (*n* = 40). Due to the adoption of the RMET ROIs (RMET Region of Interests—Molenberghs et al., 2016), the authors found that the neural regions involved in RMET are: left middle frontal gyrus, left middle temporal gyrus, and left superior frontal gyrus.

It is important to underline those two studies did not report significant differences between MCI patients and HC in TOM tasks. According to Funkiewiez et al. (2012) and Dodich et al. (2015) aMCI performances in cognitive tests used, were not significantly different from HC. Respectively, the first study assessed TOM with the ”Faux-pas Recognition test” (Stone et al., 1998; Gregory et al., 2002) on a sample of 11 aMCI. The latter used “Story-based Empathy task” (Dodich et al., 2015) on sample of 15 aMCI.

## Discussion

We found that the overall evidence of reported results in TOM tasks are in line with the literature. Most of the studies reported that TOM impairment is related to all TOM phases (mentioned before) in both affective and cognitive TOM. Those results depend on the variety of tests used by the authors to investigate multiple domains of social cognition; in particular, out of 20 articles 19 different tests were used (see Table 2). This aspect is an advantage but, on the other hand, it is a limitation because it culminated in ambiguous results; for example, some studies reported lower TOM performance while investigating only the cognitive domain of TOM (Baglio et al., 2012; Maki et al., 2013; Moreau et al., 2015), yet there are studies that showed the same results using only affective tasks (Poletti and Bonuccelli, 2013; Yildirim et al., 2020; Eramudugolla et al., 2021; Michaelian et al., 2021; Orso et al., 2022). This heterogeneity in the adoption of the tests does not allow for overlapping results. Of the 20 studies, only half of the papers use a comprehensive battery of tasks, which permits us to explore TOM in a global way and to obtain more significative results than the authors who chose to adopt a narrower battery of tasks. Despite the methodological variety, the outcomes are all in accordance; social cognition, in particular regard with TOM, seems to be significantly impaired in all types of patients with mild cognitive impairment. Another aspect to be discussed is the composition of the sample, specifically, every paper has a different type of sample based on different etiology and diagnostic system for MCI (i.e., Pertersen or DSM V). For example, some studies have a sample of amnesic MCI (Baglio et al., 2012; Maki et al., 2013), others of Parkinson's Disease -MCI (Adenzato et al., 2019; Giguère-Rancourt et al., 2022) or Alzheimer's Disease -MCI (Valera-Bermejo et al., 2021; Orso et al., 2022). Despite the results being all along the same line, it could be interesting to divide and compare the results of MCI patients based on their etiology or diagnosis. In our review, this was not achievable given the few results on the topic, but it could be a good starting point for future research. Regarding the composition of the sample, we highlight that the present review included few subjects; consequently, the outcomes obtained could not be generalized and the magnitude of an association could be overestimated.

**Table 2:** TOM tasks.

**References**	**Name**	**Brief description**
Snowden et al. (2003)	The eye-direction detection	ToM-precursor
Baron-Cohen et al. (1995)		
Perner et al. (1987)	The deceptive box task	First order false belief
Antonietti et al. (1999)	The look-prediction and say-prediction task;	Second order false belief
Sullivan et al. (1994)		
Happé (1994)	A selection from the strange stories	Complex ToM
Mazzola and Camaioni (2002)		
Sullivan and Ruffman (2004)	The reading the mind in the eyes (RMET)	Affective TOM
Castelli et al. (2011)		
Baron-Cohen (2002)		
Gregory et al. (2002)	Faux pax recognition task	Cognitive TOM
Stone et al. (1998)	(FPR)	Processing of intentions and social reasoning
Adachi et al. (2004)	Metaphoric and sarcastic scenario test	Cognitive TOM
Winner et al. (1998)	A French adaptation of the task created by Winner et al. (1998) was used in this study to evaluate verbal irony comprehension	Fact question (true/false) First order belief question. Second-order true or false belief question. Second-order belief follow-up question. Second-order expectation question. Irony or lie (I/L) comprehension question.
Achim et al. (2012)	Mentalizing abilities (Combined Stories Test) and verbal irony comprehension (SSICT)	First and second order mentalizing Non-social reasoning Attention and Memory
Champagne-Lavau et al. (2009)	The false belief task (Nosy neighbor)	True and false belief of first and second order
Samson et al. (2007)	The referential communication task	Knowledge and belief attribution
Shamay-Tsoory et al. (2007)	Italian version of the yoni task	Cognitive and Affective of first and second order
Adenzato et al. (2017)	Attribution of intentions task	Cognitive TOM
McDonald et al. (2003)	The awareness of social inference test (TASIT)	Emotion evaluation Social inferences
Dodich et al. (2015)	Story-based empathy task (intention attribution) Story based empathy task (emotion attribution)	Cognitive TOM Affective TOM
Oosterman et al. (2011)	Story comprehension task	Cognitive TOM
Molenberghs et al. (2016)	Reading the mind in the eyes test (RMET-ROIs)	Affective TOM (Regions of Interests)

Last, it is important to underline that only 2 papers out of 20 did not reported significant differences in TOM performance between MCI patients and HC (Funkiewiez et al., 2012; Dodich et al., 2015). A possible explanation of these contradictory results is that both papers have small samples and the MCI groups were not considered as the mains focus of the studies (for example, in Funkiewiez et al., 2012, aMCI patients were used just as a control group). This could explain the different results.

The most recent studies (Valera-Bermejo et al., 2021; Orso et al., 2022), in addition to investigating the TOM through a cognitive approach, adopted also a neurological approach that described the neural substrate associated with low performance in TOM. These authors discovered that low performances in social cognition were correlated with lower connectivity of the default-mode-network (DMN—i.e., a set of brain regions in temporal, parietal, and frontal cortex involved in attention and complex cognition tasks, such as abstract thought; Smallwood et al., 2021) with the prefrontal cortex. Specifically, the prefrontal cortex is the damaged brain area in MCI patients. In conclusion, DMN is highly associated with TOM performance; this evidence provides a further demonstration of what was reported in most studies mentioned before (Table 2).

## Conclusion

This systematic review analyzed the last 10 years of literature on theory of mind in MCI patients. MCI was diagnosed in several ways because there are different etiologies that could predict it (i.e., PD-MCI or AD-MCI). This could indicate a gap in the case of comparisons between studies.

Also, every author used different types of tasks to assess TOM (which measure all phases of TOM and both cognitive and affective TOM); this gave us a comprehensive view on this topic, but, on the other hand, this could be a limit if we make a comparison between results.

Our goal was to collect all results but align the results using the same diagnostic criteria, but the same type of tests could be advisable for future research.

Another limit of the sample was the small size for most of the studies and, consequently, the outcomes obtained could not be generalized and the magnitude of an association could be over-estimated.

Last, the neuropsychological approach should be more deeply explored because it could be another interesting point of view that might support the majority of results obtained up to now.

## Data availability statement

The original contributions presented in the study are included in the article/supplementary materials, further inquiries can be directed to the corresponding author/s.

## Author contributions

LS carried out part of the literature search, collected part of the studies, described part of the results, contributed to complete the table, and reviewed the final version manuscript. LM had the main contribution in the literature search and selection, created the draft, the two tables and the flow diagram, deals with defining and writing the method, wrote the abstract, the introduction and part of the results, of the discussion and the conclusion, and reviewed the references and the final version of the manuscript. AI elaborated the table and wrote part of the introduction, of the results, of the discussion and of the conclusion and reviewed the references and the final version of the manuscript. SR collected part of the literature search. MC and LR-C contributed to the collection and selection of the literature. GZ and EB contributed to the collected part of the literature search. All authors contributed to the article and approved the submitted version.

## Funding

Open access funding provided by Università Della Svizzera Italiana.

## Conflict of interest

The authors declare that the research was conducted in the absence of any commercial or financial relationships that could be construed as a potential conflict of interest.

## Publisher's note

All claims expressed in this article are solely those of the authors and do not necessarily represent those of their affiliated organizations, or those of the publisher, the editors and the reviewers. Any product that may be evaluated in this article, or claim that may be made by its manufacturer, is not guaranteed or endorsed by the publisher.
